# Gadolinium-enhanced cardiovascular magnetic resonance: administered dose in relationship to united states food and drug administration (FDA) guidelines

**DOI:** 10.1186/1532-429X-14-18

**Published:** 2012-02-29

**Authors:** Marcelo S Nacif, Andrew E Arai, Joao AC Lima, David A Bluemke

**Affiliations:** 1 Radiology and Imaging Sciences - National Institutes of Health Clinical Center, Bethesda, MD, USA; 2 Division of Cardiology, Johns Hopkins University School of Medicine, Baltimore, MD, USA; 3 Radiology Department, Universidade Federal Fluminense, Niterói, RJ, Brazil; 4 Cardiovascular and Pulmonary Branch, National Heart, Lung, and Blood Institute - National Institutes - Bethesda, MD, USA; 5 Molecular Biomedical Imaging Laboratory, National Institute of Biomedical Imaging and Bioengineering, Bethesda, MD, USA

**Keywords:** Contrast media, Gadolinium, Heart, Magnetic resonance imaging

## Abstract

**Purpose:**

Myocardial late gadolinium enhancement was originally validated using higher than label-recommended doses of gadolinium chelate. The objective of this study was to evaluate available evidence for various gadolinium dosing regimens used for CMR. The relationship of gadolinium dose warnings (due to nephrogenic systemic fibrosis) announced in 2008 to gadolinium dosing regimens was also examined.

**Methods:**

We conducted a meta-analysis of peer reviewed publications from January, 2004 to December, 2010. Major subject search headings (MeSh) terms from the National Library of Medicine's PubMed were: contrast media, gadolinium, heart, magnetic resonance imaging; searches were limited to human studies with abstracts published in English. Case reports, review articles, editorials, MRA related papers and all reports that did not indicate gadolinium type or weight-based dose were excluded. For all included references, full text was available to determine the total administered gadolinium dose on a per kg basis. Average and median dose values were weighted by the number of subjects in each study.

**Results:**

399 publications were identified in PubMed; 233 studies matched the inclusion criteria, encompassing 19,934 patients with mean age 54.2 ± 11.4 (range 9.3 to 76 years). 34 trials were related to perfusion testing and 199 to myocardial late gadolinium enhancement. In 2004, the weighted-median and weighted-mean contrast dose were 0.15 and 0.16 ± 0.06 mmol/kg, respectively. Median contrast doses for 2005-2010 were: 0.2 mmol/kg for all years, respectively. Mean contrast doses for the years 2005-2010 were: 0.19 ± 0.03, 0.18 ± 0.04, 0.18 ± 0.10, 0.18 ± 0.03, 0.18 ± 0.04 and 0.18 ± 0.04 mmol/kg, respectively (p for trend, NS). Gadopentetate dimeglumine was the most frequent gadolinium type [114 (48.9%) studies]. No change in mean gadolinium dose was present before, versus after the Food and Drug Administration (FDA) black box warning (p > 0.05). Three multi-center dose ranging trials have been published for cardiac MRI applications.

**Conclusion:**

CMR studies in the peer-reviewed published literature routinely use higher gadolinium doses than regulatory agencies indicated in the package leaflet. Clinical trials should be supported to determine the appropriate doses of gadolinium for CMR studies.

## Background

Cardiovascular Magnetic Resonance (CMR) is a highly reproducible modality able to assess myocardial tissue characteristics and myocardial perfusion [1,2]. The most common gadolinium (Gd) enhanced techniques for CMR are detection of myocardial scar [3,4] using late gadolinium enhancement (LGE) methods and detection of myocardial ischemia using stress/rest perfusion CMR [5-7].

CMR provides outstanding characterization of myocardial size and function, but LGE is a unique capability of CMR compared to other imaging modalities. LGE CMR was originally validated using higher than label-recommended doses of gadolinium (Gd) chelate [3,8-10], i.e., 0.2 mmol/kg of a conventional gadolinium chelate.

In 2006, the association between nephrogenic systemic fibrosis (NSF) and exposure to Gd-based contrast agents (GBCAs) was reported [11]. Although multiple different associations with NSF and GBCA have been reported, the most important are renal failure and dialysis [12,13]. In late 2007, the United States Food and Drug Administration (FDA) issued a black box warning regarding GBCAs due to its association to NSF [14]. European regulators also issued warnings at the same time. All GBCAs had revised label warnings in early 2008. Centers performing magnetic resonance imaging (MRI) instituted policies to identify patients at high risk for NSF [13], including advanced age (> 60 yrs), eGFR < 30 mL/min/1.73 m^2^, dialysis and acute renal failure. The success of a screening policy for NSF was recently demonstrated, further suggesting that renal failure and high dose gadolinium administration may be the primary underlying risk factors for NSF development [15]. Recently in 2010, the FDA further recommended that label doses of GBCA's (generally 0.1 mmol/kg for "conventional" GBCAs) not be exceeded in any patient.

Currently, gadolinium enhanced MRI of the heart is off-label use for all FDA approved GBCAs. The purpose of this is to evaluate evidence-based dosing regimens for GBCAs for CMR. In particular, we describe CMR dosing regimens used before and after the FDA black box warnings that were instituted in 2008.

## Methods

We conducted a meta-analysis on MEDLINE of peer reviewed publications from January, 2004 to December, 2010. Full text was evaluated for all matching references to determine the total administered gadolinium dose. The publications were examined for potentially duplicate or overlapping data. Corresponding investigators were contacted for clarification when data were unclear or inadequate. QUOSA Information Manger Version 8.0 (QUOSA Inc., Waltham, MA, USA) was used to search and retrieve full text publications using appropriate major subject search headings (MeSh) terms from the National Library of Medicine's PubMed abstract collection. The MeSh terms were: contrast media, gadolinium, heart, magnetic resonance imaging. Searches were limited to human studies with abstracts published in English from January 1, 2004 to December 3, 2010.

Peer-reviewed publications were included in the analysis if: 1) stress/rest perfusion or LGE were performed of the myocardium; 2) the number and age of study participants were reported; 3) gadolinium dose was reported on a per kilogram (kg) basis. Studies that described only phantoms or animals were excluded. Case reports, review articles, editorials, and magnetic resonance angiography (including coronary angiography) studies were excluded. Full text was available for all studies. Studies that evaluated both perfusion *and *LGE were classified as LGE studies. For those studies, the total GBCA dose was used for analysis.

### Statistical analysis

Categorical variables are presented as numbers and percentages. Median and mean values were weighted according to the number of subjects in each study. The main analysis was performed at the patient level, and the secondary analysis was performed at the contrast media level. Two-sample two tailed t-test was used to determine significant differences between two sets of results. P-values < 0.05 were considered to be statistically significant.

## Results

**Figure 1:**
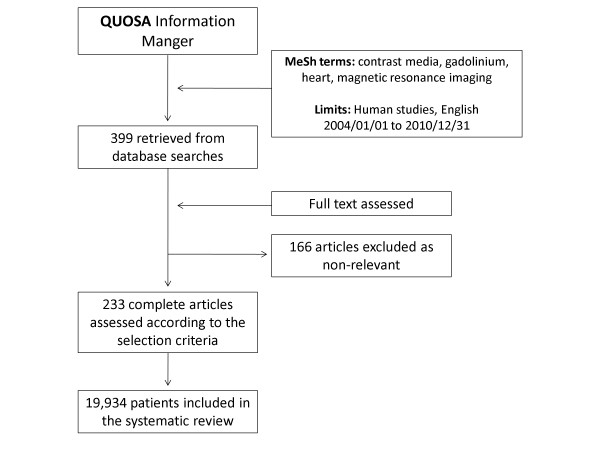
**Flow diagram of review identification and selection of patients include meta-analysis**.

PubMed searches identified 399 potentially relevant publications. Table 1 summarizes the Current FDA approved GBCAs. One hundred sixty-six studies were excluded. The exclusion causes are summarized in Table 2. Two hundred thirty-three studies matched the inclusion criteria encompassing 19,934 patients with mean age 54.2 ± 11.4 (range 9.3 to 76 years) in the peer-reviewed literature published between 2004 and 2010. The majority of the patients (11,793, 59.2% were between 41 and 60 years of age. Four hundred seventeen (2%) patients were less than 20 years old, 1,565 (7.9%) patients were between 21 and 40 years, and 6,158 (30.9%) of patients were older than 60 years. The literature selection process is summarized in Figure 1.

**Table 1:** Current FDA approved GBCAs

Contrast agent	Trade name	Manufacture	Label dose	Age	FDA Approved indication
Gadopentetate Dimeglumine (Gd-DTPA2)	Magnevist^®^	Bayer Healthcare	0.1 mmol/Kg (0.2 mL/Kg)	2 years and older	Central Nervous System* Extracranial/Extraspinal Tissues** Body (excluding the heart)***
Gadodiamide (Gd-DTPA-BMA)	Omniscan^®^	GE Healthcare	0.1 mmol/Kg (0.2 mL/Kg) 0.05 mmol/Kg (0.1 mL/Kg)§	2-16 years and adults	Central Nervous System* Body [noncardiac]****
Gadoversetamide (Gd-DTPA-BMEA)	OptiMark^®^	Mallinckrodt	0.1 mmol/Kg (0.2 mL/Kg)	18 to 76 years	Central Nervous System* Liver
Gadoteridol	ProHance^®^	Bracco Diagnostics	0.1 mmol/Kg (0.2 mL/Kg) Additional dose of 0.2 mmol/Kg (0.4 mL/Kg) §§§	2 - 18 years and adults	Central Nervous System* Extracranial/Extraspinal Tissues**
Gadobenate Dimeglumine (Gd-BOPTA)	MultiHance^®^	Bracco Diagnostics	0.1 mmol/Kg (0.2 mL/Kg)	2 years and older	Central Nervous System*
Gadobutrol (Gd-DO3A-butrol)	Gadavist^®^ Gadovist^®^;§§	Bayer Healthcare	0.1 mmol/Kg (0.1 mL/Kg)	2 years and older	Disrupted Blood Brain Barrier Central Nervous System*
Gadofosveset trisodium	Ablavar^®^ Vasovist^®^§§	Lantheus Medcl	0.03 mmol/Kg (0.12 mL/Kg)	Adults	Aortoiliac occlusive disease
Gadoxetate disodium	Eovist^®^ Primovist^®^§§	Bayer Healthcare	0.025 mmol/Kg (0.1 mL/Kg)	Adults	Liver Lesions

*Outside the US only, shown for completeness*					
*Gadoterate Meglumine (Gd-HP-DOTA)*	*Dotarem^®^* *Artirem^®^§§*	*Guerbet*	*0.1 mmol/Kg (0.2 mL/Kg)*	*2 years and older*	Central Nervous System* Body [noncardiac]****

Other agents previously approved but not currently available are Mangafodipir Trisodium (Teslascan^®^; GE Healthcare) and Ferumoxides (Feridex^®^; Amag Pharms). NDA, New Drug Application. * Abnormal vascularity in the brain (intracranial lesions), spine and associated tissues. ** Abnormal vascularity in the head and neck. *** Abnormal vascularity in the body. **** Intrathoracic, abdominal, pelvic cavities, and the retroperitoneal space. § For Kidney studies. §§ Only for use outside the United States. §§§ May be given up to 30 minutes after the first dose for patients with normal renal function suspected of having poorly enhancing central Nervous System lesions.

**Table 2:** Reasons for exclusion of publications

	2004	2005	2006	2007	2008	2009	2010	Total
Phantom or animal model	0 (0)	2 (8)	5 (20)	4 (21)	5 (15.6)	6 (17.7)	4 (21)	26 (15.7)
No weight-based contrast dose	2 (16.7)	9 (36)	5 (20)	3 (15.8)	6 (18.7)	8 (23.5)	1 (5.2)	34 (20.4)
Case reports	3 (25)	3 (12)	5 (20)	3 (15.8)	7 (21.9)	8 (23.5)	5 (26.4)	34 (20.4)
Review articles	1 (8.3)	2 (8)	2 (8)	5 (26.4)	7 (21.9)	6 (17.7)	4 (21)	27 (16.3)
Editorials	4 (33.3)	5 (20)	4 (16)	0 (0)	0 (0)	2 (5.8)	0 (0)	15 (9.1)
MRA	2 (16.7)	4 (16)	4 (16)	4 (21)	7 (21.9)	4 (11.8)	5 (26.4)	30 (18.1)

Total	12 (100)	25 (100)	25 (100)	19 (100)	32 (100)	34 (100)	19 (100)	166 (100)

MRA, magnetic resonance angiography. Data is presented as number and percentages.

**Figure 2:**
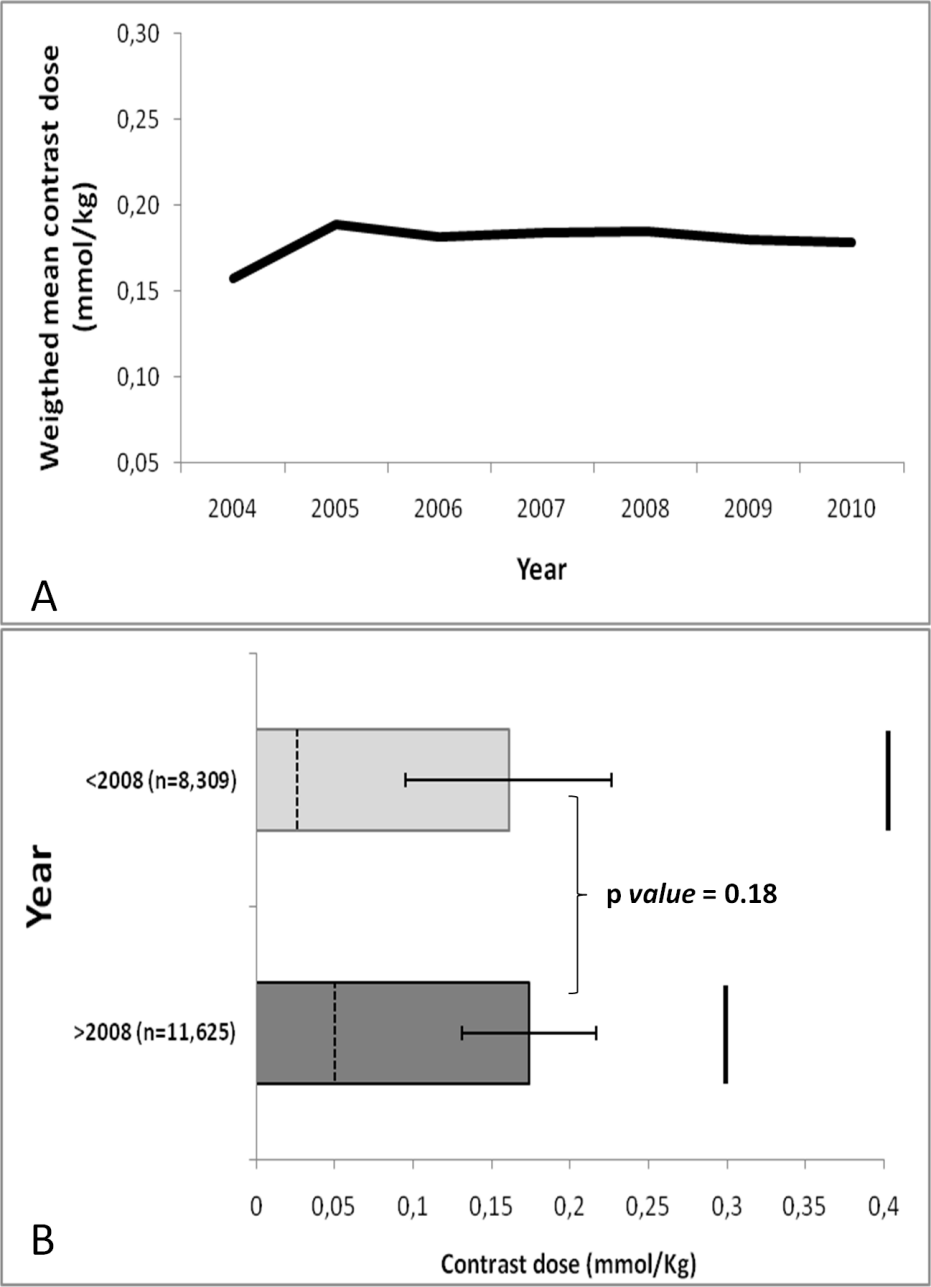
**(A) Weighted mean contrast dose (mmol/Kg) from 2004 to 2010**. (**B**) No change in mean gadolinium contrast dose before versus after FDA black box warning. Dashed horizontal lines represents minimum contrast dose and horizontal lines represents maximum contrast dose.

Study and population characteristics are summarized in Table 3. Thirty-four trials were classified as perfusion evaluation and 199 were myocardial LGE evaluation. The most common clinical applications were myocardial infarction and viability testing (n = 88; 37.7%) followed by cardiomyopathy (n = 76; 32.6%) studies (Table 4). Ischemia evaluation accounted for 14.5% of the publications. The main focus of the publication was related to pre and post-surgery of congenital heart disease in 8 studies (3.4%). In 2004, the weighted-median and -mean reported contrast dose was 0.15 and 0.16 ± 0.06 mmol/kg, respectively. Median contrast doses for 2005-2010 were: 0.2 mmol/kg for all years, respectively. Mean contrast doses for the years 2005-2010 were: 0.19 ± 0.03, 0.18 ± 0.04, 0.18 ± 0.10, 0.18 ± 0.03, 0.18 ± 0.04 and 0.18 ± 0.04 mmol/kg, respectively (p for trend, not significant). No change in mean gadolinium dose was present before, versus after the FDA black box warning (p > 0.05) (Figure 2).

**Table 3:** Characteristics of publications included in the meta-analysis

Year	Studies	Participants	Age (years)	Perfusion studies	LGE studies	Median GBCA dose (mmol/Kg)*	Mean GBCA dose (mmol/Kg)*
2004	22 (9.5)	697 (3.5)	56.1 ± 8.7	6 (17.6)	16 (8.1)	0.15	0.16 ± 0.06
2005	19 (8.2)	2,123 (10.7)	54.4 ± 8.4	2 (5.9)	17 (8.6)	0.2	0.19 ± 0.03
2006	26 (11.1)	4,366 (22.0)	54.9 ± 11.3	5 (14.8)	21 (10.5)	0.2	0.18 ± 0.04
2007	31 (13.3)	1,123 (5.6)	46.1 ± 16.9	3 (8.9)	28 (14.1)	0.2	0.18 ± 0.10
2008	40 (17.1)	2,264 (11.3)	55 ± 8.9	7 (20.5)	33 (16.5)	0.2	0.18 ± 0.03
2009	45 (19.3)	3,965 (19.9)	56.2 ± 10.3	5 (14.7)	40 (20.1)	0.2	0.18 ± 0.04
2010	50 (21.5)	5,396 (27.0)	55 ± 11.3	6 (17.6)	44 (22.1)	0.2	0.18 ± 0.04

Total	233 (100)	19,934 (100)	54.2 ± 11.4	34 (100)	199 (100)	0.2	0.18 ± 0.04

Median, Mean and standard deviations or number and percentages as appropriate. LGE, late gadolinium enhancement.

Studies that had both LGE and perfusion results were categorized as LGE. For these studies, total gadolinium dose is shown.

**Table 4:** Topics of CMR studies that used GBCAs in peer reviewed publications

	2004	2005	2006	2007	2008	2009	2010	Total
Myocardial infarction	6 (27.2)	3 (15.8)	9 (34.6)	5 (16.1)	5 (12.5)	17 (3.7)	16 (32)	61 (26.1)
Others cardiomyopathies*	1 (4.6)	6 (31.5)	4 (15.4)	10 (32.2)	11 (27.5)	10 (22.3)	10 (20)	52 (22.3)
Ischemia	6 (27.2)	2 (10.5)	5 (19.2)	3 (9.6)	7 (17.5)	5 (11.2)	6 (12)	34 (14.5)
Myocardial viability	5 (22.7)	3 (15.8)	2 (7.6)	1 (3.3)	6 (15)	4 (8.9)	6 (12)	27 (11.6)
Hypertrophic Cardiomyopathy	2 (9.1)	4 (21.1)	1 (3.9)	2 (6.5)	5 (12.5)	3 (6.6)	7 (14)	24 (10.3)
Myocarditis	1 (4.6)	0 (0)	0 (0)	1 (3.3)	0 (0)	0 (0)	0 (0)	2 (1.0)
Pulmonary Hypertension	1 (4.6)	1 (5.3)	0 (0)	1 (3.3)	0 (0)	2 (4.3)	0 (0)	5 (2.1)
Valvular disease	0 (0)	0 (0)	2 (7.7)	0 (0)	1 (2.5)	1 (2.3)	1 (2)	5 (2.1)
Cardiac mass	0 (0)	0 (0)	1 (3.9)	3 (9.6)	1 (2.5)	0 (0)	0 (0)	5 (2.1)
Others	0 (0)	0 (0)	2 (7.7)	5 (16.1)	4 (10)	3 (6.7)	4 (8)	18 (7.9)

Total	22 (100)	19 (100)	26 (100)	31 (100)	40 (100)	45 (100)	50 (100)	233 (100)

Data is presented as number and percentages. * Heart Failure, Post-Surgery status, Deposit disease, ARVD, Atrial Fibrilation, Congenital Heart disease, Tokotsubo

Over all years, 4678 participants received gadolinium in perfusion protocols. For perfusion, the weighted-median and -mean contrast doses were 0.2 and 0.17 ± 0.05 mmol/Kg, respectively. Also, 15,256 participants were studied in LGE protocols. For LGE MRI, the weighted-median and -mean contrast doses were 0.2 and 0.18 ± 0.04 mmol/Kg (p < 0.001 compared to mean dose for perfusion studies).

From 2004 to 2010, Gadopentetate dimeglumine (Magnevist^®^) was used most frequently (n = 114; 48.9% of the publications) followed by Gadodiamide (Omniscan^®^) (n = 39; 16.7%) and Gadoterate meglumine (Dotarem^®^) (n = 15; 6.5%). Gadoterate meglumine is not currently available in the United States. A large percentage of trials reported incomplete or inconsistent data regarding type and dose of GBCAs, (23.1% of all studies). Table 5 summarizes those findings.

**Table 5:** Use of gadolinium contrast agents for CMR studies in peer reviewed publications

Contrast agent	Publications (percent) by year							
	2004	2005	2006	2007	2008	2009	2010	Total
Gadopentetate Dimeglumine	13 (59)	10 (52.6)	11 (42.3)	15 (48.3)	24 (60)	25 (55.5)	16 (32)	114 (48.9)
Gadodiamide	4 (18.2)	4 (21)	5 (19.2)	8 (25.8)	4 (10)	5 (11.1)	9 (18)	39 (16.7)
Gadoterate Meglumine	0 (0)	0 (0)	2 (7.7)	4 (13)	0 (0)	5 (11.1)	4 (8)	15 (6.5)
Gadobutrol	0 (0)	0 (0)	0 (0)	0 (0)	3 (7.5)	0 (0)	2 (4)	5 (2.2)
Gadobenate Dimeglumine	0 (0)	0 (0)	0 (0)	1 (3.2)	1 (2.5)	1 (2.3)	1 (2)	4 (1.8)
Gadoversetamide	0 (0)	0 (0)	1 (3.8)	0 (0)	1 (2.5)	0 (0)	0 (0)	2 (0.8)
Not specified	5 (22.8)	5 (26.4)	7 (27)	3 (9.7)	7 (17.5)	9 (20)	18 (36)	54 (23.1)

Total	22 (100)	19 (100)	26 (100)	31 (100)	40 (100)	45 (100)	50 (100)	233 (100)

Data is presented as number and percentages

Table 6 shows multicenter phase II/III CMR studies that have been performed for assessment of GBCA dose. One study evaluated gadolinium dose for LGE (566 patients). The other ones (99 and 94 patients, respectively) evaluated GBCA dose for myocardial perfusion.

**Table 6:** Prior Multi-Center Phase II/III studies on GBCAs for CMR indications

Investigators	Number of study subjects	Year	Journal	Protocol	Contrast agent	mmol/kg	Recommended gadolinium dose
Wolff et al [17]	99	2004	Circulation	Perfusion	Gadopentetate Dimeglumine	0.05; 0.10; 0.15	0.05 mmol/kg
Giang et al [18]	94	2004	Eur Heart J	Perfusion	Gadopentetate Dimeglumine	0.05; 0.10; 0.15	0.10 or 0.15 mmol/Kg
Kim et al [16]	566	2008	Circulation	LGE	Gadoversetamide	0.5, 0.1, 0.2 or 0.3	0.2 mmol/kg

## Discussion

Given the recent attention to the potential adverse effects of high dose gadolinium MRI studies, we evaluated the current status of GBCA use for CMR as presented in the peer-reviewed literature, emphasizing trends before and after nephrogenic fibrosis guidelines were issued in 2008. This meta-analysis showed that the median GBCA dose for English peer reviewed publications for CMR (19,934 patients) was 0.2 mmol/kg. Further, no change in mean or median gadolinium dose was present before, versus after the FDA issued GBCA black box warnings (p > 0.05). To date, only 1 multi-center, dose ranging trial has been conducted for a single gadolinium contrast agent (gadoversetaminde) for LGE [16]. For perfusion CMR, two dose ranging multi-center trials for gadopentetate dimeglumine have been published in the literature, with "optimal" gadolinium dose ranging from 0.05 to 0.15 mmol/kg [17,18], The gadolinium dose reported in the literature for perfusion CMR varies between 0.1-0.2. (Table 6) [16-18]. It remains to be seen if future CMR studies will incorporate and report lower gadolinium doses.

CMR is widely available and has been validated at 0.2 mmol/Kg for detection of scar [3,4] and at a range of doses for myocardial ischemia using stress/rest perfusion protocol [5-7]. The most frequent topics in the peer-reviewed literature focused on myocardial infarction [19,20] and viability [21,22], cardiomyopathy [23-25], ischemia [26,27], and myocarditis [28]. It seems quite clear that these published, investigational studies have routinely used higher doses of GBCAs than FDA labeling. A limitation of this report is that we are unable to determine if the pattern of GBCA use with CMR in *published *studies reflects the broader routine clinical pattern of practice for GBCA use.

Patients with cardiovascular disease must be screened for risk factors that are associated with NSF. In addition to the major risk factor of renal failure, an additional risk factor for NSF might to be multiple/high dose gadolinium enhanced MRI examinations. In particular, Adujudeh et al [29-31], Perez et al [15] and others have shown that patients who receive higher cumulative doses of gadopentetate dimeglumine have a higher risk of developing NFS compared with those who received lower doses. Patients who have cardiovascular disease may have other medical conditions that require MRI examination. Thus, the relatively high dose of GBCA used for CMR studies may be particularly relevant for patients who may also undergo non-cardiovascular MRI.

The accepted approach to determination of the minimally effective GBCA dose is evaluation in multi-center phase II and III studies. For LGE, only one contrast agent (gadoversetamide) has been subject to a combined phase II/III evaluation. That study concluded that a gadolinium dose of 0.2 mmol/kg gadoversetamide was appropriate for LGE CMR. For myocardial perfusion, Wolff et al indicated that the appropriate dose for a single perfusion evaluation was 0.05 mmol/kg with gadopentetate dimeglumine [17]. Giang et al showed that gadolinium doses of 0.10 or 0.15 of gadopentetate dimeglumine were optimal for perfusion CMR. Since that time however, considerable advances have been made in both hardware and software.

Approximately 23% of peer-reviewed publications regarding gadolinium enhanced CMR had incomplete or inaccurate information related to GBCAs. "Gadolinium DTPA," a nonspecific description of the specific contrast agent, was frequently described as the type of contrast media that was administered. In order to maintain patient safety, it is essential that gadolinium contrast agents be accurately reported in the medical record. Contrast agents for MRI are currently treated the same as all other hospital medications by the United States Joint Commission on Accreditation of Healthcare Organizations. These standards require accurate recording and reconciliation of the type, route and dose of administration. The American College of Radiology standards for the practice of MRI also indicates that radiology reports should record type, route and dose of gadolinium administration. With the advent of NSF, it is likely that improved reporting of gadolinium type may take place in the future.

Dose and efficacy studies for GBCAs focused on CMR have rarely been performed. One factor is likely the high cost of multi-center trials which is typically borne by the drug manufacturer. Thus, peer-reviewed literature and individual physician experience from single-site experience will probably continue to guide clinical practice. In principle, the lowest drug dose that is diagnostically efficacious is recommended by regulatory authorities. Without well-controlled clinical trials, the lowest gadolinium dose that is efficacious is essentially unknown for most of the available CMR contrast agents. Recently, there have been attempts to use lower gadolinium dose in large multi-center studies, such as EuroCMR (gadolinium dose < 0.16 mmol/kg) [32]. In addition, the Multi-Ethnic Study of Atherosclerosis (MESA)[33] protocol for LGE CMR involving approximately 3000 study participants requires a gadolinium dose of 0.15 mmol/kg; a higher dose of 0.2 mmol/kg was not recommended by the MESA renal working group due to safety concerns in an elderly, volunteer population (D. Bluemke, J Lima, personal communication).

It is clear that improved reporting of contrast media administration for CMR publications needs immediate attention in the peer reviewed literature. We strongly recommend that authors, editors and reviewers of peer-reviewed journals demand standardization of GBCA reporting in the literature. Professional societies that are particularly concerned about the use of CMR, such as the Society of Cardiovascular Magnetic Resonance (SCMR) and the International Society for Magnetic Resonance in Medicine (ISMRM), could provide education and guidelines for practice in this regard. For CMR, it is important to report not only the weight based dose of the GBCA (mmol/kg or ml/Kg) and the chemical name and manufacturer, but also the delay time for imaging. Generic statements such as "gadolinium DTPA" should be avoided.

There are several limitations of this study. We limited our search to articles with abstracts and those published in English with specific MeSh terms from PubMed. Thus, our review probably does not reflect the worldwide use of gadolinium contrast agents. In addition, the FDA black box warning in 2007 was issued by a United States agency. We hypothesized that CMR publications appearing after 2008 were likely to be influenced by this warning. However, warnings about gadolinium use were slightly different in Europe although they were also issued about this same time. In addition, FDA-approved GBCA medication label changes took place only in early 2008. It is possible that changes in gadolinium dose policies in research studies may take longer to appear in the literature than our inclusion literature date of December, 2010. The observation of discernable trend in dose reduction for CMR over this period might be a reflection of changing referral patterns for CMR: patients with renal failure are no longer being referred for GBCA studies and there is less consideration for low dose by CMR physicians in a somewhat healthier population.

In conclusion, we report that CMR studies in the peer-reviewed published literature routinely use higher gadolinium doses than FDA label indicated dose. Clinical trials should be supported to determine the appropriate doses of gadolinium enhancement of the myocardium.

## Competing interests

The authors declare that they have no competing interests.

## Authors' contributions

MSN: study design, data acquisition, data analysis, data interpretation, manuscript revision; AA: study design, manuscript revision; JL: study design, manuscript revision; DB principal investigator, study design, data interpretation, manuscript revision.

All authors read and approved the final manuscript.
